# Advances in spatiotemporal models for non-communicable disease surveillance

**DOI:** 10.1093/ije/dyz181

**Published:** 2020-04-15

**Authors:** Marta Blangiardo, Areti Boulieri, Peter Diggle, Frédéric B Piel, Gavin Shaddick, Paul Elliott

**Affiliations:** 1 UK Small Area Health Statistics Unit; 2 MRC-PHE Centre for Environment & Health, Department of Epidemiology & Biostatistics, Imperial College London, London, UK; 3 Centre for Health Informatics, Computing and Statistics (CHICAS), Lancaster Medical School, Lancaster University, Lancaster, UK; 4 Department of Mathematics, University of Exeter, Exeter, UK

**Keywords:** Surveillance, non-communicable diseases, Bayesian hierarchical models, spattemporal modelling

## Abstract

Surveillance systems are commonly used to provide early warning detection or to assess an impact of an intervention/policy. Traditionally, the methodological and conceptual frameworks for surveillance have been designed for infectious diseases, but the rising burden of non-communicable diseases (NCDs) worldwide suggests a pressing need for surveillance strategies to detect unusual patterns in the data and to help unveil important risk factors in this setting. Surveillance methods need to be able to detect meaningful departures from expectation and exploit dependencies within such data to produce unbiased estimates of risk as well as future forecasts. This has led to the increasing development of a range of space-time methods specifically designed for NCD surveillance. We present an overview of recent advances in spatiotemporal disease surveillance for NCDs, using hierarchically specified models. This provides a coherent framework for modelling complex data structures, dealing with data sparsity, exploiting dependencies between data sources and propagating the inherent uncertainties present in both the data and the modelling process. We then focus on three commonly used models within the Bayesian Hierarchical Model (BHM) framework and, through a simulation study, we compare their performance. We also discuss some challenges faced by researchers when dealing with NCD surveillance, including how to account for false detection and the modifiable areal unit problem. Finally, we consider how to use and interpret the complex models, how model selection may vary depending on the intended user group and how best to communicate results to stakeholders and the general public.

**Key Messages**

- There is increasing recognition of the importance of surveillance for NCDs.
- Spatiotemporal variation in health outcomes and lifestyle and environmental exposures needs to be explicitly modelled in order to reduce bias and uncertainty.
- Hierarchical modelling provides a coherent framework within which spatiotemporal dependencies can be explicitly modelled with integration of the uncertainties associated with both the data and the modelling process.
- In a simulation study, we found that mixture models designed for detection perform better than standard disease mapping models. However, attention should be paid to the choice of threshold, as this affects the results. It is recommended that a simulation study based on the characteristics of the data in hand is run each time for the selection of suitable threshold values.
- Current research challenges in this area include: the use of data from multiple sources at different spatial and temporal scales and with different sources of bias and uncertainty; computationally intense processes; and control for falseipositive findings.

## The Importance of Non-communicable Diseases

According to the World Health Organization (WHO), surveillance is the ‘ongoing systematic data collection, analysis and interpretation and dissemination of information in order for action to be taken’.^1^ National public health agencies, such as the US Centers for Disease Control and Prevention (CDC) and Public Health England (PHE), routinely carry out surveillance data analysis to provide early warnings of unexplained changes in incidence patterns of diseases as well as to aid policy formation and resource allocation.^2^ Specific examples include the international influenza monitoring system which started in 1948 and is now distributed in 82 countries,^3^ the HIV and AIDS Reporting System (HARS) used by PHE^4^ and the National HIV Surveillance System used by CDC.^4^^,^^5^

To date, the majority of methods and models commonly used in public health surveillance are designed for monitoring cases of infectious diseases.^6^ Due to the rising burden of non-communicable diseases (NCDs) worldwide, there is a pressing need to implement surveillance strategies to detect trends, highlight unusual changes and consequently assist in outlining emerging NCD risk factors. NCD surveillance shares many objectives with infectious disease surveillance, including generating information to guide public health action and detecting the health impact of environmental exposures or of environmentally driven disease vectors; however, it also presents some different methodological challenges.^7^^,^^8^

Health data contain both a time and a space component. Surveillance methods must be able to capture spatial and temporal patterns in both lifestyle/environmental exposures and health outcomes. Here, we present an overview of the approaches developed for spatiotemporal disease surveillance of NCDs. We focus on model-based methods and, among these, on Bayesian hierarchical models (BHMs) which can naturally accommodate complex data structures, as well as propagate uncertainty due to the data themselves and the modelling process.

In this section, we first discuss how data availability is one of the key challenges in surveillance studies, before giving a generic overview of test-based approaches for NCD surveillance. We then focus on BHMs and describe disease mapping and mixture-based models for anomaly detection. Later we introduce the computational aspects of the BHM modelling framework for NCD surveillance; then we run a simulation study to evaluate advantages and drawbacks of the approaches presented in detecting areas deviating from the expected trend. Finally, in the Discussion, we conclude with a summary and some remaining discussion points.

### Data availability

One of the major challenges of surveillance studies is the availability of suitable data. This applies to both infectious disease and NCD surveillance. It is a particularly important issue in low-income settings because surveillance studies often need to rely on information from surveys, and the lack of financial resources may make comprehensive coverage of data sources (e.g. mortality/cancer registries) over an entire country infeasible.^9^

In the past 15 years, a number of Health and Demographic Surveillance Systems (HDSS) have been established in low-income settings to provide a reliable source of health data, and are now linked together through the International Network for the continuous Demographic Evaluation of Populations and Their Health (INDEPTH^10^). Such continuous surveys are an invaluable source of data, but researchers face issues related to population representativeness. A recent study proposed a Bayesian probabilistic clustering method to evaluate the network representativeness in terms of socioeconomic and environmental variables in sub-Saharan Africa, identifying areas of poor coverage in the existing network and using predictive probability distributions to suggest the best location for new HDSS sites.^11^

Even in high-income settings where administrative resources are available for the entire population, there may be issues regarding the population at risk, used as denominator in the risk estimates. In small-area studies on mortality or hospital admissions, the denominator is usually the resident population in each administrative area, typically estimated from national census statistics, but there may be estimation problems for intercensual years. In addition, it is not straightforward to define the denominator where the interest is for less-defined geographies, such as the catchment areas of clinical centres (e.g. general practices in England).

Furthermore, the availability of administrative or health data may become more limited: for instance, within the UK National Health Service, patients can now decide not to share their medical records for research purposes. This clearly impacts on spatial coverage and could potentially lead to biased statistical inference if the data gaps are clustered in space and/or if they differentially affect specific population groups (e.g. elderly, more deprived).^12^

### Test-based methods

Methods for NCD surveillance have largely been based around the idea of detecting whether the outcome of interest shows a particular behaviour in a defined subset (e.g. an area, period of time, or combination of space and time) when compared with the whole study region. Perhaps the most popular test-based methods used for NCD surveillance are the scan statistics. These were developed originally in the temporal setting only^13^; here, a fixed length ‘scanning window’ is passed over the time-series data with the number of cases in the window being recorded. A log likelihood ratio (LLR)^14^ is calculated for each interval, and the test statistic is defined as the maximum LLR over all intervals. This idea was extended to a spatial version of the scan statistic,^15^ which was later further extended to the spatiotemporal setting.^16^ In this case, the scanning window is represented by a cylinder, where the diameter specifies the spatial dimension and the height the temporal dimension. An additional version of spatial scan statistic was proposed to account for correlation across spatial units, which was not considered before.^17^ Scan statistics have been extensively applied to numerous health care applications. Part of their popularity lies in the availability of free user-friendly SaTScan^TM^ software [https://www.satscan.org/]. Recent applications of SaTScan include the identification of signals for colorectal cancer,^18^ drug activity,^19^ criminality^20^ and bat activity.^21^

A further development has been the detection of spatial variations in temporal trends (SVTT). These methods extend the scan statistics to estimate the time trend via a regression-based model specifying either a linear or a quadratic function. The quadratic SVTT method has, for example, been applied to cervical cancer data in women in the USA from 1969 to 1995, highlighting areas where the risk was significantly different from the rest.^22^

Test-based methods such as scan statistics can only answer questions related to the deviation from the null hypothesis. An alternative approach is to explicitly model the spatiotemporal structure in the data and to assess whether differences between observed data and those predicted from the model provide evidence of anomalies. There are a number of advantages to adopting a model-based approach over a test-based method, including the ability to: (i) have more statistical power to handle sparsity in the observed disease counts; (ii) explore more subtle departures from the expectation; (iii) account for the spatial and temporal correlation that is typically evident in health data; (iv) ‘borrow’ information over space and time, therefore increasing the precision of the estimates generated; and (v) include covariates that might explain some of the spatiotemporal variability.

## Hierarchical Models and Likelihood-based Inference

Hierarchical models (HM) are able to deal with complex data structures, to exploit dependencies between data sources and to propagate the inherent uncertainties that are present in both the data and the modelling process. In the current context, an HM combines two elements: a process model that describes how disease risk varies over space and time, typically involving both extant covariate effects and a latent spatiotemporal stochastic process; and a data model that describes the statistical properties of the available health outcome data conditional on the realization of the underlying risk process. Both elements are specified up to the values of a set of unknown parameters, which can be estimated by Bayesian or non-Bayesian versions of likelihood-based inference, typically implemented using Markov chain Monte Carlo integration and Monte Carlo likelihood maximization methods, respectively. In addition to estimating parameters, the scientific goals of health surveillance include prediction of relevant properties of the unobserved risk surface as it evolves in real time. Parameters and latent stochastic processes are fundamentally different things, but within the Bayesian paradigm they are both treated as unobserved sets of random variables, and the operational calculus of estimation and prediction coalesces. In what follows, we use BHM as a shorthand for Bayesian inference applied to a hierarchically specified model.

### Space-time disease mapping

A class of BHMs which has been extensively used for the analysis of NCD data comprises the so-called disease mapping models (DM). These are hierarchical models in which the latent component of area-level disease risk is modelled as a spatially discrete Markov random field^23^ and, depending on the sampling design, the conditional distribution of area-level case-counts is Poisson or Binomial.^24–27^ Whereas the objectives of these are descriptive, they have been used as the basis for the development of detection models, which are framed in a surveillance perspective. Disease mapping models have been extensively used to estimate and visualize the spatial or spatiotemporal distribution of a disease (see for instance Diggle and Giorgi, Adam and Fenton, and WHO^9^^,^^21^^,^^28^).

Spatial dependence in the latent component of a DM is modelled by specifying neighbourhood relationships among the area-level risks, the most widely used definition being that two areas are neighbours if they share a common boundary. A common choice for capturing temporal dependence is a random walk prior,^29^ but extensions to incorporate spatiotemporal interactions among neighbouring areas and time points have also been developed.^30^ This framework can also account for factors known to modify spatial and temporal trends that, in the context of NCDs, will include demographic variables (e.g. age/sex/ethnicity) and social economic status. Random effects can be assigned for each factor (with appropriate priors) and for interactions if required. An example is provided by Goicoa *et al*.^31^ who proposed a space-time-age model to study prostate cancer incidence across 50 provinces in Spain for nine age groups over 25 years, accounting for all pair-wise interactions. The authors used ranking of all provinces according to mortality rates to identify high-risk groups.

A key characteristic of BHMs is the ready availability of joint posterior/predictive distributions for parameters/latent processes and whatever of their properties are relevant to the public health questions of interest. In the context of disease mapping, this leads to a spatial, temporal and spatiotemporal risk distribution that researchers can map, in terms of point estimates but also of associated measures of uncertainty. For the latter, a common choice is the predictive probability that the relative risk exceeds a prespecified threshold.^32^^,^^33^ Exceedance probabilities can be used to flag areas and/or time points characterized by increased risk that may then be further investigated. In this way disease mapping, though not formally a surveillance method, can be used as a descriptive tool for the identification of areas and/or time periods with marked deviation from expectation. It is important to note that the strong smoothing effect of disease mapping models leads to conservative risk estimates, hence to a small number of false-positive findings, at the expense of a low power for detecting high-risk areas with low signal. To minimize that, an extensive simulation was run to find the best threshold on the exceedance probability scale to classify an area as high risk.^33^ The authors showed that a good trade-off between false-positive and false-negative rates is achieved with a probability above 0.8 for a relative risk to be higher than 1; however, this largely depends on the number of expected counts, the number of areas and time points and the spatial risk.^34^

**Figure 1.**
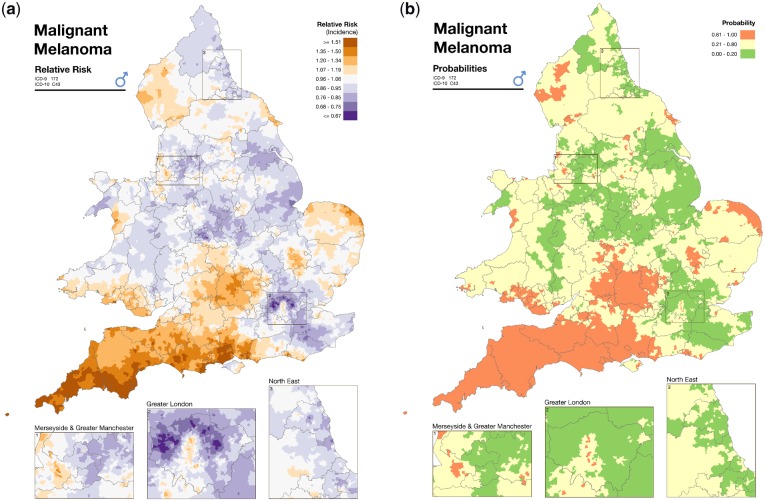
(a) Area-specific posterior mean relative risk of malignant melanoma. Source: *Environment and Health Atlas.*^35^ (b) Area-specific posterior probability that an area is characterized by a relative risk of malignant melanoma above 1. Source: *Environment and Health Atlas*.^42^.

As an example of the typical disease mapping output, Figure 1 shows the incidence of malignant melanoma in males, at the census ward level in England and Wales over the period 1985–2009, from the Environmental and Health Atlas produced by the UK Small Area Health Statistics Unit (SAHSU).^36^ The map on the left presents the spatial distribution of the posterior relative risk mean estimates, and the map on the right plots the posterior probability that the corresponding relative risk is above 1, using the categorization suggested in Richardson *et al*.^33^

Disease mapping models can be extended to two or more outcomes that might share spatial (and temporal) patterns, for instance due to common risk factors. A joint model allows information to be borrowed across the outcomes, thus helping stabilize estimates, particularly when the outcomes are rare. The shared component model,^37^ originally developed for two diseases, includes a common component (likely to reflect common risk factors) and a disease-specific one, which can point towards specific risk factors otherwise masked in a single disease model. It was applied to male and female lung cancer^38^ and later extended to jointly model multiple diseases,^30^^,^^39^ with an application on oral cavity, oesophagus, larynx and lung cancers in males in the 544 districts of Germany from 1986 to 1990. Recently, it was further extended to jointly model age- and gender-specific diseases.^40^

An alternative multivariate specification considers spatial and temporal terms explicitly, modelling the correlation among the outcomes in space/time. As an example, road traffic accidents characterized by different severity were analysed over the period 2005–11 at the ward level in England while detection of high-risk areas was performed using exceedance probabilities of the area ranks based on accident rates.^41^

### Space-time anomaly detection

The standard disease mapping approach has been used informally to detect anomalies (unusual observations) in space and time, i.e. areas and/or time points with trends different from the expected ones, adding a space-time interaction parameter into the latent process.^38^ The detection of anomalies may indicate the presence of an emerged localized risk factor, the impact of an intervention, or differences in the quality of data, such as misdiagnosis of a disease, and under- or over-reporting of cases.

Mixture models have been proposed as a formal approach to anomaly detection. In particular, Abellan *et al.*^42^ developed a BHM model (termed STmix) where a mixture of two normal distributions characterized by different variances is specified for the space-time interaction. Then, the interaction is used to classify areas as common or as unusual. The authors performed a simulation study to compare the method against the standard disease mapping approach. The results of the simulation study showed that the standard approach was not able to capture the variability in the spatiotemporal interactions and therefore it was not able to distinguish between common and unusual areas. This is due to the excessive smoothing following the assumption of a common variance across all the areas and time points. STmix was applied to mammography screening data in Brisbane, Australia, at the statistical local area (SLA) level from 1997 to 2008, in order to identify SLAs whose temporal trend exhibited volatility.^35^ A well-known drawback of this approach is its limitation in incorporating specific time patterns, for example step changes that could signal the emergence of a new risk factor.

Another mixture model, proposed by Li *et al*.,^43^ accommodates this issue. Here, the mixture specification of the method is defined directly on the relative risks in space and time, to allow for detection of areas with unusual time trends rather than space-time deviations. In particular, two alternative models are considered: the first one assumes a global time trend for all areas (common trend), and the second estimates a time trend for each area independently (area-specific trend). Through a simulation study, the authors showed better performance in terms of both sensitivity and proportion of false-positives compared with SaTScan, on a wide range of scenarios. This approach, named BaySTDetect, was applied to detect unusual trends for asthma and chronic obstructive pulmonary disease at Clinical Commissioning Group (CCG) level in England (211 in total) on monthly data between August 2010 and March 2011, across mortality, hospital admissions and general practice drug prescriptions.^44^

**Figure 2.**
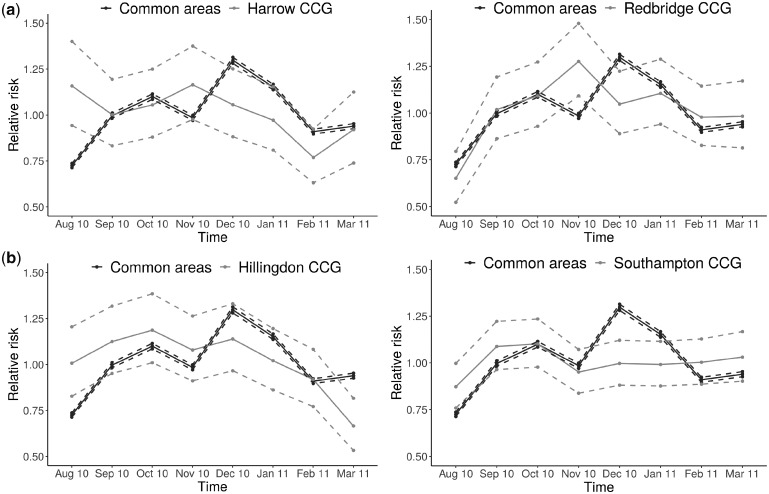
(a) Relative risks and 95% credible intervals of hospital admissions for asthma and COPD for the national (common) temporal trend and for Harrow CCG, classified as unusual. (b) Relative risks and 95% credible intervals of hospital admissions for asthma and COPD for the national (common) temporal trend and for Hillingdon CCG, classified as unusual.

To illustrate the typical output obtained from this model, Figure 2 shows the area-specific time trends of the CCGs which were detected as unusual, plotted against the national trend. Other applications of this method include burglary data,^45^ grey whale abundance^46^ and mammography data.^35^

This method was further extended to increase its flexibility by accounting for different space-time patterns in the unusual observations, as well as by allowing for longer time series to be analysed. This improved method, termed FlexDetect, had a better performance when compared with the original method through an extensive simulation study.^47^

### Multiple testing

As surveillance studies involve evaluating trends for different health outcomes, many areas and different time periods at the same time, false detections are likely to occur by chance. Bonferroni correction has been extensively used in epidemiology to correct for multiple testing, particularly in omics studies,^48^^,^^49^ but it is well known that this approach leads to conservative results. Benjamini and Hochberg first introduced an alternative index, the false-discovery rate (FDR)^50^ as the expected value of the rate of false-positive findings among all rejected hypotheses, and used it in a frequentist approach. The same method was suggested in the context of descriptive spatial epidemiology, to obtain areas characterized by a Standardised Mortality Ratio different from 1.^51^ Even in the Bayesian setting, FDR rules were suggested by many authors.^52–56^ The mixture model proposed by Li *et al*. uses the specification suggested by Newton *et al*.,^53^ later used by Ventrucci *et al*.^57^ in order to account for multiple testing. The authors base the FDR statistic on the posterior model probability, which represents the likelihood of the space-time unit investigated to follow the common trend model, i.e. to exhibit a risk pattern not deviating from the expected one.^43^

Whereas the importance of controlling for multiple testing is clear in classical significance testing, the analogous problem in predictive setting is less of a concern.^58^ One reason for this is that local predictions from hierarchical models are naturally smoothed towards the global mean, making these consequently less prone to false-positive findings than unsmoothed area-by-area interval estimates. Another is that the Monte Carlo sampling method allows the computation of whatever joint probability statements are required. For example, if the public health question is whether current risk exceeds an agreed acceptable level in all areas that do, and in no areas that do not, meet a particular criterion such as adherence to a particular advisory policy, the correct predictive probability to attach to this statement can be calculated.

## Computational Aspects

One of the biggest challenges researchers face when analysing large and complex space-time datasets is their computational burden. This applies particularly in the small-area context, where the number of space-time units investigated can vary substantially depending on the chosen spatial and temporal resolution, from a few hundreds to hundreds of thousand units, particularly when several outcomes are jointly analysed (for instance, Foreman *et al*.^59^ considered jointly deaths/age/sex specific space-time trends in the USA).

The degree of complexity of the model (e.g. the number of parameters) also impacts on the computational burden, for instance in terms of convergence time when running MCMC simulations. Under the Bayesian paradigm, the choice of the prior will also influence convergence; an informative prior, assuming that there is no conflict with the data, will normally speed up convergence, whereas a vague prior will most likely lead to longer time to reach convergence. Finally, the choice of software used for the analysis will affect the model running time. The user-friendly software BUGS (Bayesian inference Using Gibbs Sampling)^60^ has been traditionally used for Bayesian inference using MCMC methods; however, it can be slow when high-dimensional data and/or complex models are used. Other MCMC-based methods, such as Stan^61^ and NIMBLE,^62^ are currently attracting attention due to their active development community. An alternative way of dealing with computational limitations is to use approximative methods; for instance INLA (Integrated Nested Laplace Approximations)^63^ has been successfully used for running space-time disease mapping models (e.g.^31^^,^^64^) however, this method is somewhat less flexible than the aforementioned ones and, as it relies on Normality of the latent process, is not able to deal with mixture distributions.

Computationally intensive BHMs benefit from high-performance computing clusters to speed up computation times, but these are not necessarily required. For instance in road traffic accidents,^41^ data of different severity in England were analysed simultaneously at the ward level (∼8000) over 9 years (for a total of around 150 000 units and 32 000 parameters); the analysis was run in OpenBUGS and took 20-27 h on an Intel Core processor at 3.40 GHz with 8 Gbytes of random-access memory. On a much bigger scale, a US small-area study considered more than half a million units and nearly 6000 parameters^59^; the analysis was implemented in Stan using higher performance computing (HPC) clusters for faster calculations.

## Simulation-based Example

Considering the multitude of space-time methods available as described above, it is important to formally evaluate their respective detection performance. In this paper we carried out a simulation study to formally evaluate the detection performance and compare DM, STmix and FlexDetect (see description of the models in Space-time disease mapping). Following the design initially proposed by Li *et al*.,^43^ and later used by Boulierei *et al*.,^47^ we used real asthma hospital episode statistics (HES) data to generate 50 simulated datasets. The asthma dataset was obtained from SAHSU, Imperial College London, and consisted of disease counts across 211 Clinical Commissioning Groups (CCG) in England for 15 months, from January 2010 to March 2011. The 50 simulated datasets were generated to closely resemble the patterns seen in the real dataset. A standard spatiotemporal model^27^ was first fitted to the real data, and the obtained parameters were selected for the generation of the simulated data. We selected 15 areas to deviate from the overall time trend over the last five time points. For these 15 areas, we selected the signal to be increased by log(2) for time points 3 and 10, and decreased by log(2) for time points 6, 12 and 15 out of the total 15 time points. In this way, we ensured that a realistic scenario was used (for more details see Boulieri *et al*.,^47^ and Supplementary materials, Scenario 1, available as Supplementary data at *IJE* online). The R code used for the data simulation, together with the three models written in BUGS, can be found on [https://github.com/aretib/bayesSTmodels.git].

The results are presented in Table 1 in terms of four different performance measures. We defined TP as the number of true-positives, FP as false-positives, TN as true-negatives and FN as false-negatives, respectively. Sensitivity measures the ability of the model to correctly classify an unusual observation as such, defined as TP/(TP+FN), and similarly specificity measures the ability of the model to correctly classify a common observation as such [TN/(TN+FP)]. In addition, it is crucial to control the proportions of observations that are falsely classified as unusual [false-discovery rate (FDR)] and common [false omission rate (FOR)], respectively; these should typically not exceed a value of 0.05, specified based on the standard *P*-value threshold. We consider: (i) two different thresholds for DM: 0.8 as commonly used and previously described (DM1): a more conservative threshold of 0.9 (DM2), under the assumption that false-positives are more important to minimize than false-negatives; and (ii) two different rules for STmix as presented in the original paper: an area is modified if at least for one time point the space-time interaction has a probability greater than 0.8 to be above 1 (STmix1); an area is modified if for at least three time points the space-time interactions have an average probability greater that 0.8 to be above 1 (STmix2).

**Table 1.** Posterior mean and 95% credible intervals for the competing models in the simulation study. We compared the detection performance of disease mapping (DM1, DM2), the mixture model on the spatiotemporal interaction (STmix1, STmix2) and the mixture model on the spatiotemporal rates (FlexDetect)

	FDR	FOR	Sensitivity	Specificity
DM1	0.785	0.002	0.979	0.722
	(0.773, 0.800)	(0.000, 0.006)	(0.933, 1.000)	(0.695, 0.744)
DM2	0.191	0.026	0.660	0.987
	(0.100, 0.267)	(0.020, 0.030)	(0.600, 0.733)	(0.981, 0.995)
STmix1	0.000	0.017	0.773	1.000
	(0.000, 0.000)	(0.010, 0.024)	(0.683, 0.867)	(1.000, 1.000)
STmix2	0.220	0.002	0.969	0.978
	(0.167, 0.300)	(0.000, 0.005)	(0.933, 1.000)	(0.969, 0.985)
FlexDetect	0.019	0.005	0.796	1.000
	(0.015, 0.031)	(0.004, 0.006)	(0.763, 0.827)	(0.999, 1.000)

As can be seen, the disease-mapping approach using the standard threshold of 0.8 on the posterior probability scale (DM1) shows the worst performance. As expected, the method is able to detect nearly all unusual areas, with a sensitivity of 0.979; however, roughly 79% of the detected findings are not actually unusual (FDR = 0.785) (Table 1). Fixing a 0.9 threshold (DM2), FDR decreases, despite still being above the standard threshold of 0.05, while at the same time sensitivity also decreases (0.660) (Table 1).

The two mixture models returned more comparable performances. STmix1 gave no false-positive results (FDR = 0) and a sensitivity of 0.773, whereas for STmix2 sensitivity increased to 0.969, but at the same time a much higher proportion of false-positives was detected (FDR = 0.220). The results of FlexDetect provided a balance between the two extremes, giving a proportion of FDR equal to 0.019 and a sensitivity of 0.796. In terms of specificity and FOR, both STmix and FlexDetect behave similarly.

Differences across the competing models were observed in terms of computation time, an important factor in assessing their performance. All models were run in an Intel Xeon Core processor 3.40 GHz with 125GB RAM. Each of the 50 simulations took on average 33.4 min for models DM1 and DM2, 39.2 min for models STmix 1 and STmix2 and 66.8 min for FlexDetect. The simulation results suggest that using disease mapping (DM) for surveillance purposes is not appropriate and that one of the mixture models designed for detection should be used instead. Between STmix and FlexDetect, it is worth mentioning that STmix can only identify areas where anomalies are present, and not the time points when these occur. In addition to this, its detection mechanism does not consider specific patterns in the time trends. These can be accommodated by FlexDetect, which however is more computationally intensive. Also note that mixture models notoriously have problems converging, suffering from issues such as label switching, which lead to multimodal posterior distributions. Both the detection methods deal with this through the modelling specification,^42^ constraining the variance of the modified areas to be larger than that of unmodified areas, or through informative priors on the variances of the two components.^43^

## Discussion

In this paper we have presented an overview of the main statistical methods for disease surveillance in the context of NCDs, both from a test-based and model-based perspective and with a particular focus on the BHM approach, which provides a flexible framework to allow for complex data dependencies present in surveillance studies. Through a simulation study we showed that disease mapping is not satisfactory when looking for data anomalies, whereas the two methods based on mixture models provide a better compromise between detecting areas characterized by a deviation from the expected trend and limiting false-positives. Note that our perspective is on methods that detect single areas, rather than clusters of adjacent spatial units. If the interest lies in detection in the presence of spatial proximity, recent methods have been developed to combine clustering with spatial smoothing, see for example Anderson *et al*.^65^ and Adin *et al*.^66^

An interesting aspect of the general hierarchical framework presented is that it can easily incorporate forecasting of the disease risk, which is relevant in the context of epidemiological surveillance to evaluate the need for resources/policies/costs in specific areas and at future time points. Some work in this area includes Foreman *et al*.^59^ who, using annual vital statistics for 1974–2011 at the US state spatial resolution, forecasted mortality up to 2024; and Ugarte *et al*.^67^ used P-splines to forecast cancer mortality counts in Spanish regions for 2009–11 using data from 1975–2008.

Most of the work presented is based on routinely collected data for retrospective studies. However, there is increased importance of early warning detection, so that unusual behaviour can be detected at the earliest possible time. Syndromic data, such as primary care data, drug prescriptions, nurse calls and home visits, which are indicative of a potential anomaly, may provide an additional level of information leading to a detection event before the data aberration occurs.^68^ Diggle *et al*.^69^^,^^70^ analysed NHS non-emergency telephone calls reporting symptoms of gastrointestinal diseases. The authors specified a spatiotemporal point process on the location and time of the individual calls and modelled the spatial and temporal dependency on the intensity of the process. They used exceedance probabilities to define maps of potential outbreaks. Another example can be found in Morrison *et al*.^71^ who forecasted multiple measures of healthcare use (including physician visits and prescriptions of asthma medication) within British Columbia, Canada, where seasonal wildfires produce high levels of air pollution, significantly affecting population health. Here, the focus was on efficient, near real-time, computation which was achieved using INLA to perform approximate Bayesian inference.

Potentially syndromic information can also be linked with routine data such as Hospital Episode Statistics and can provide predictors in order to obtain a better description of the data and more accurate one-step-ahead forecasts. Lately work has been done to take advantage of the rich data from social media in a surveillance perspective. For instance, Dai *et al.*^72^ linked tweets with the American Community Survey and the Behavioral Risk Factor Surveillance System to study asthma prevalence at the state level in the USA. The authors claimed that the inclusion of social media data could be a cost-effective real-time health detection system. However, there may be challenges in future due to selective data availability following perceived concerns about data security and confidentiality, as demonstrated by the newly implemented NHS National Data Optout Programme. This will potentially lead to bias in population representativeness due to non-random missingness^12^ which will need to be addressed using advanced statistical methods, for instance through the integration of data from appropriate surveys/cohorts, as proposed in the context of residual confounding.^73^

An important issue with surveillance studies is that of the spatial resolution and the type of geographical areas considered; modifying these might lead to different results, as the spatial distribution of the outcome will depend on these choices. For instance, if within-area variability is substantial, results from statistical inference might suffer from false-negative observations, as potentially high-risk places are aggregated with low-risk ones. The more spatial variability is present in the data, the more profound the potential impact of the modifiable areal unit (MAUP).^74^^,^^75^ As MAUP depends on the level of aggregation, this issue has been linked to ecological bias,^76^ and the general suggestion in the scientific literature is to consider the finest spatial scale available. This can be particularly challenging for rare diseases where the numbers of cases at small-area level are very low. Furthermore, the choice of spatial resolution is mostly dependent on data availability and sparsity. BHMs have been suggested as a way to, at least partially, deal with MAUP. As there is an explicit relationship among areas globally and/or locally, through structured random effects, places belonging to a particularly small-area can influence results for other areas, hence alleviating the MAUP problem.^77^

A key aspect of surveillance studies concerns how to communicate information to public health researchers and policy makers. This is particularly challenging as the statistical modelling of surveillance data becomes more sophisticated. In this context it is essential to develop user-friendly tools such as atlases, web applications and reporting services, which allow for data visualizations and easy implementation of the advanced methodologies. The Environment and Health Atlas for England and Wales^36^ (typical output from the Atlas was presented in Figure 1) is an example of work in this direction, providing stakeholders and the general public with a collection of maps to inform on the spatial distribution of environmental factors and diseases. Through the exceedance probabilities, these maps give a perception of the uncertainty around the area-level relative risks estimates.

Web applications allow the ready implementation of statistical methods and perform complex data analyses, often through interactive data visualizations. These can be particularly useful for practitioners less skilful in statistical modelling and programming. As an example, the Rapid Inquiry Facility (RIF) which is currently being redeveloped within SAHSU, is designed to facilitate disease mapping and risk analysis studies and has been employed by more than 45 institutions in a number of countries.^78^ A more recent example is the SpatialEpiApp that integrates two methods for disease mapping and cluster detection.^79^

To conclude, in this paper we presented a range of BHMs, which have proved to be useful for non-communicable disease surveillance. The choice of model should depend on various factors and, most importantly, on the objective of the study, characteristics of the data, and computational resources. It is commonly recommended to perform simulation studies based on the data in hand, to inform the model and to select detection rules that are most appropriate in each case.

We believe that epidemiological surveillance will be at the centre of future methodological research to match the continuous increase in data availability, e.g. through social media; this will also open up issues related to data integration, selection bias and spatiotemporal misalignment. At the same time there will be the need to reduce the computational burden of increasingly complex models applied to large datasets, in order to provide timely results for decision making.

## Funding

The UK Small Area Health Statistics Unit (SAHSU) is part of the MRC-PHE Centre for Environment and Health, which is supported by the Medical Research Council (MR/L01341X/1) and Public Health England (PHE). Part of this work was supported by an Early Career MRC-PHE Fellowship awarded to A.B. and a Wellcome Trust Seed Award in Science awarded to F.B.P. (204535/Z/16/Z). P.E. is Director of SAHSU and Director of the MRC-PHE Centre for Environment and Health.

**Conflict of interest:** None declared.
